# Crystal structure of 5-[4-(di­ethyl­amino)­benzyl­idene]-2,2-dimethyl-1,3-dioxane-4,6-dione

**DOI:** 10.1107/S2056989015017673

**Published:** 2015-09-26

**Authors:** Egija Stepina, Dmitrijs Stepanovs, Inese Mierina, Mara Jure

**Affiliations:** a Faculty of Materials Science and Applied Chemistry, Riga Technical University, Str. P. Valdena 3/7, Riga, LV 1048, Latvia; b Latvian Institute of Organic Synthesis, Str. Aizkraukles 21, Riga, LV 1006, Latvia

**Keywords:** crystal structure, aryl­idene Meldrum’s acid, 5-aryl­methyl­ene-2,2-dimethyl-1,3-dioxan-4,6-dione, organic synthesis, intra­molecular hydrogen bonding

## Abstract

The title compound, C_17_H_21_NO_4_, consists of substituted Meldrum’s acid with a [4-(di­ethyl­amino)­phen­yl]methyl­idene fragment attached to the fifth position. The heterocycle assumes a distorted boat conformation. The planar part of heterocycle is almost coplanar with the benzene ring due to the presence of a long conjugated system in the mol­ecule. This leads to the formation of C—H⋯O-type intra­molecular contacts. As a result of the absence of hydrogen-bond donors in the structure, the crystal packing is controlled by van der Waals forces and weak C—H⋯O inter­actions, which associate the mol­ecules into inversion dimers.

## Abstract 2

The title compound, 5-[4-(di­ethyl­amino)­phenyl­methyl­idene]-2,2-dimethyl-1,3-dioxane-4,6-dione, have been synthesized and its crystal structure determined. Due to the absence of hydrogen-bond donors in the structure, the crystal packing is controlled by van der Waals forces and weak C—H⋯O inter­actions, which associate the mol­ecules in dimers.

## Chemical context   

Aryl­idene Meldrum’s acids (5-aryl­methyl­idene-2,2-dimethyl-1,3-dioxane-4,6-diones) are attractive building blocks in organic chemistry: these compounds are used for the synthesis of different heterocycles. Recent examples include: pyrazolidinones (Pair *et al.*, 2014 ▸), lactames (Zhang *et al.*, 2013 ▸), carbocycles (*e.g*. Trost & Maruniak, 2013 ▸) and aliphatic compounds (*e.g*. Mohite & Bhat, 2013 ▸). Aryl­idene Meldrum’s acids can be easily converted to aryl­methyl Meldrum‘s acids [for a description of a typical procedure, see Mierina *et al.* (2015 ▸)], which serve as starting compounds for the synthesis of various valuable compounds [for a mini-review, see Mierina (2014 ▸)]. Apart from their wide application in syntheses, these derivatives of Meldrum’s acid have been studied as platelet aggregation inhibitors (El Maatougui *et al.*, 2012 ▸), anti­malarial agents and anti-oxidants (Sandhu *et al.*, 2010 ▸) and photostable UV-filters for cosmetic applications (Habeck & Krause, 1999 ▸).
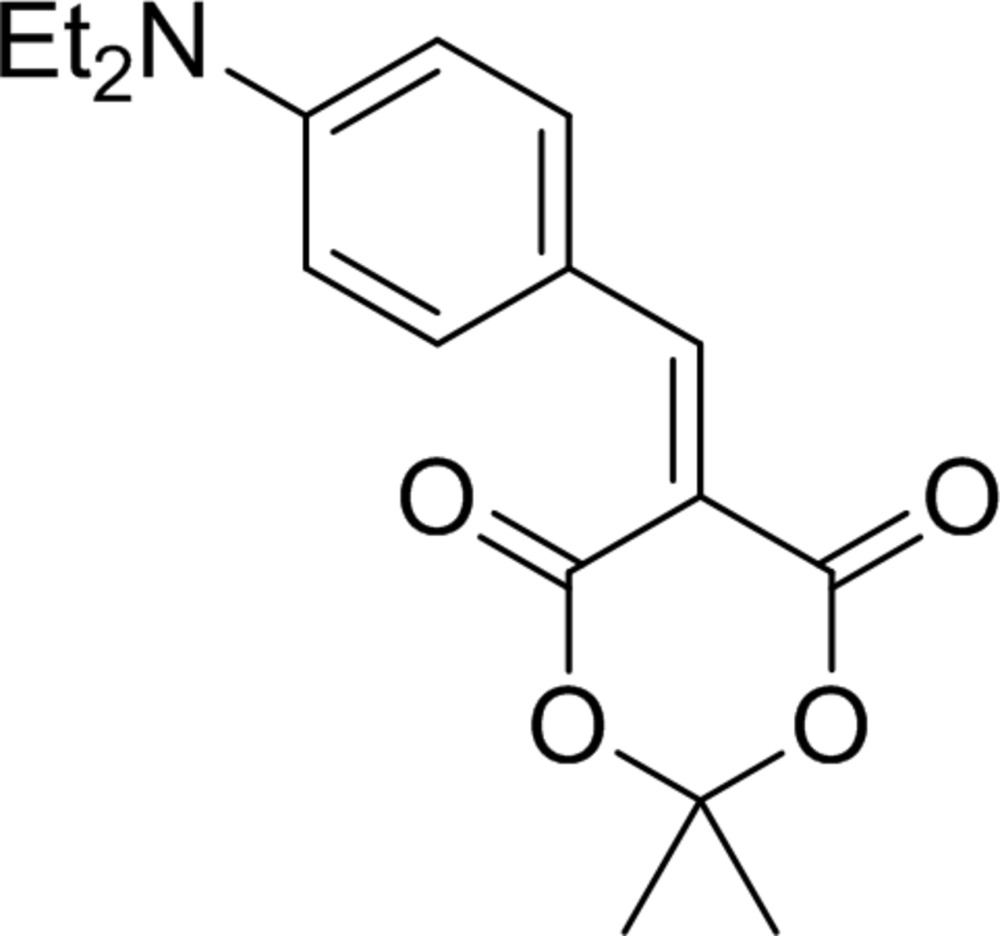


## Structural commentary   

**Figure 1:**
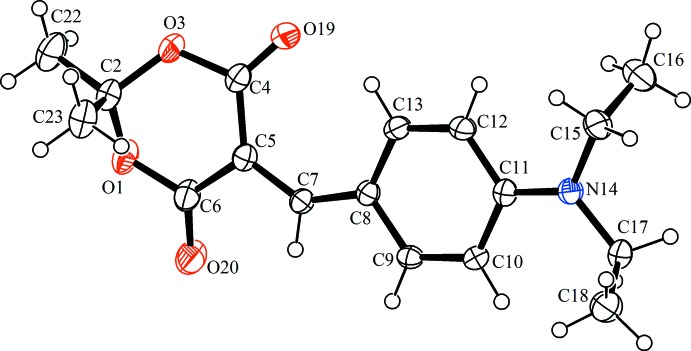
The mol­ecular structure the title compound, showing 50% probability displacement ellipsoids and the atomic numbering

The title compound, C_17_H_21_NO_4_, consists of substituted Meldrum’s acid with a [4-(di­ethyl­amino)­phen­yl]methyl­idene fragment attached to fifth position (Fig. 1 ▸.). The heterocycle assumes a distorted boat conformation. Atoms C2 and C5 deviate from the least-squares plane [maximum deviations ±0.013 (1) Å] calculated for the other four atoms of the heterocycle by 0.549 (3) and 0.154 (3) Å, respectively. The planar part of heterocycle is nearly coplanar with the benzene ring [dihedral angle = 8.05 (10)°] due to the presence of a long conjugated system in the mol­ecule. This leads to the formation of C—H⋯O-type intra­molecular contacts (Table 1 ▸).

π–π stacking inter­actions are also observed between conjugated systems of the mol­ecules. The distance between the corresponding least-square planes is 3.54 **(su?)** Å.

The crystal structure of the zwitterionic form of 5-[4-(di­eth­ylamino)­benz­yl]-2,2-dimethyl-1,3-dioxane-4,6-dione has been already reported (Mierina *et al.*, 2015 ▸). The title compound differs from this by the presence of a double bond between atoms C5 and C7.

## Supra­molecular features   

**Figure 2:**
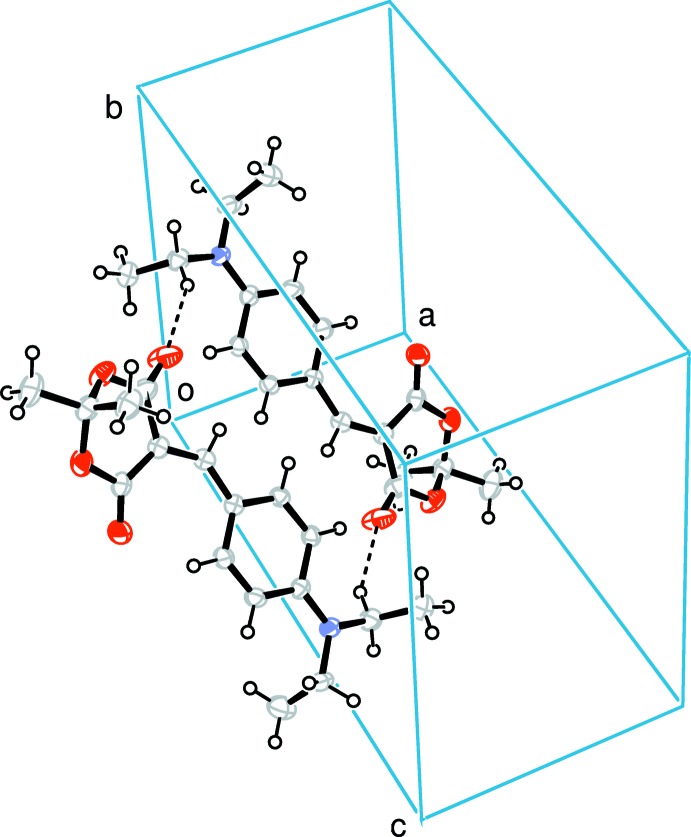
The crystal packing of the title compound, viewed along the *b* axis. Hydrogen bonds are shown as dashed lines (see Table 1 ▸ for details).

Because of the absence of hydrogen-bond donors in the structure, the crystal packing is controlled by van der Waals forces and weak C—H⋯O inter­actions, which associate mol­ecules into inversion dimers (Fig. 2 ▸, Table 1 ▸).

## Database survey   

Several 5-aryl­idene-2,2-dimethyl-1,3-dioxane-4,6-diones (Huck *et al.*, 1995 ▸; Gould *et al.*, 1998 ▸; Novoa de Armas *et al.*, 2000 ▸; O’Leary *et al.*, 2001 ▸; O’Leary & Wallis 2006 ▸; Crawford & McNab, 2009 ▸; Wilsily & Fillion, 2009 ▸; Zeng, 2010*a* ▸,*b* ▸, 2011*a* ▸,*b* ▸,*c* ▸, 2013 ▸; Jie, 2012 ▸; García-Álvarez *et al.*, 2013 ▸; Dey *et al.*, 2015 ▸) and their spiro-analogues (Sato *et al.*, 1989 ▸; Zeng, 2011*d* ▸,*e* ▸,*f* ▸; Zeng *et al.* 2013 ▸) have been characterized by X-ray analysis. However, information on the crystal structure of 5-aryl­methyl­idene-2,2-dimethyl-1,3-dioxane-4,6-diones containing an amino functionality on the aromatic ring is not available.

## Synthesis and crystallization   

5-[4-(Di­ethyl­amino)­phenyl­methyl­idene]-2,2-dimethyl-1,3-dioxane-4,6-dione was obtained from Meldrum’s acid (1.00 g, 6.9 mmol) and 4-di­ethyl­amino­benzaldehyde (1.27 g, 6.9 mmol) by heating in water (50 ml) at 348 K for 2 h, followed by cooling to room temperature and filtration of the formed precipitate and recrystallization from ethanol (1.62 g, 80%) analogously to the method described previously (Mierina *et al.*, 2015 ▸). The spectroscopic and physical data correspond to those in the literature (Mierina *et al.*, 2015 ▸). X-ray quality single crystals were obtained by slow evaporation from ethanol.

## Refinement   

Crystal data, data collection and structure refinement details are summarized in Table 2 ▸. The C-bound H atoms were positioned geometrically and refined as riding on their parent atoms: C—H = 0.93–0.98Å with *U*_iso_(H) = 1.5*U*_eq_(C) for methyl H atoms and 1.2*U*_eq_(C) for other H atoms.

## Supplementary Material

Crystal structure: contains datablock(s) I, global. DOI: 10.1107/S2056989015017673/xu5872sup1.cif

Structure factors: contains datablock(s) I. DOI: 10.1107/S2056989015017673/xu5872Isup2.hkl

Supporting information file. DOI: 10.1107/S2056989015017673/xu5872Isup3.cml

CCDC reference: 1426237

Additional supporting information:  crystallographic information; 3D view; checkCIF report

## Figures and Tables

**Table 1:** Hydrogen-bond geometry (, )

*D*H*A*	*D*H	H*A*	*D* *A*	*D*H*A*
C13H13O19	0.93	2.13	2.915(2)	141
C17H17*B*O20^i^	0.97	2.39	3.268(3)	151

Symmetry code: (i) 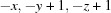.

**Table 2:** Experimental details

Crystal data	
Chemical formula	C_17_H_21_NO_4_
*M*_r_	303.35
Crystal system, space group	Monoclinic, *P*2_1_/*c*
Temperature (K)	173
*a*, *b*, *c* ()	7.8662(2), 11.4601(3), 18.1517(6)
()	96.858(1)
*V* (^3^)	1624.62(8)
*Z*	4
Radiation type	Mo *K*
(mm^1^)	0.09
Crystal size (mm)	0.26 0.19 0.09

Data collection	
Diffractometer	Nonius KappaCCD
No. of measured, independent and observed [*I* > 2(*I*)] reflections	6627, 3705, 2183
*R*_int_	0.054
(sin /)_max_ (^1^)	0.649

Refinement	
*R*[*F* ^2^ > 2(*F* ^2^)], *wR*(*F* ^2^), *S*	0.055, 0.127, 1.00
No. of reflections	3705
No. of parameters	203
H-atom treatment	H-atom parameters constrained
_max_, _min_ (e ^3^)	0.18, 0.19

Computer programs: *KappaCCD Server Software* (Nonius, 1997 ▸), *HKL* *DENZO* and *SCALEPACK* (Otwinovski Minor, 1997 ▸), *SIR2011* (Burla *et al.*, 2012 ▸), *ORTEP-3 for Windows* (Farrugia, 2012 ▸), *SHELXL97* (Sheldrick, 2008 ▸), *PLATON* (Spek, 2009 ▸) and *publCIF* (Westrip, 2010 ▸).

## supplementary crystallographic information

### Crystal data

**Table:**

C_17_H_21_NO_4_	*F*(000) = 648
*M**_r_* = 303.35	*D*_x_ = 1.240 Mg m^−^^3^
Monoclinic, *P*2_1_/*c*	Mo *K*α radiation, λ = 0.71073 Å
Hall symbol: -P 2ybc	Cell parameters from 15405 reflections
*a* = 7.8662 (2) Å	θ = 1.0–27.5°
*b* = 11.4601 (3) Å	µ = 0.09 mm^−^^1^
*c* = 18.1517 (6) Å	*T* = 173 K
β = 96.858 (1)°	Plate, red
*V* = 1624.62 (8) Å^3^	0.26 × 0.19 × 0.09 mm
*Z* = 4	

### Data collection

**Table:**

Nonius KappaCCD diffractometer	2183 reflections with *I* > 2σ(*I*)
Radiation source: fine-focus sealed tube	*R*_int_ = 0.054
Graphite monochromator	θ_max_ = 27.5°, θ_min_ = 2.3°
CCD scans	*h* = −10→10
6627 measured reflections	*k* = −14→13
3705 independent reflections	*l* = −23→23

### Refinement

**Table:**

Refinement on *F*^2^	Primary atom site location: structure-invariant direct methods
Least-squares matrix: full	Secondary atom site location: difference Fourier map
*R*[*F*^2^ > 2σ(*F*^2^)] = 0.055	Hydrogen site location: inferred from neighbouring sites
*wR*(*F*^2^) = 0.127	H-atom parameters constrained
*S* = 1.00	*w* = 1/[σ^2^(*F*_o_^2^) + (0.0534*P*)^2^ + 0.0774*P*] where *P* = (*F*_o_^2^ + 2*F*_c_^2^)/3
3705 reflections	(Δ/σ)_max_ < 0.001
203 parameters	Δρ_max_ = 0.18 e Å^−^^3^
0 restraints	Δρ_min_ = −0.19 e Å^−^^3^

### Special details

**Table:**

Geometry. All e.s.d.'s (except the e.s.d. in the dihedral angle between two l.s. planes) are estimated using the full covariance matrix. The cell e.s.d.'s are taken into account individually in the estimation of e.s.d.'s in distances, angles and torsion angles; correlations between e.s.d.'s in cell parameters are only used when they are defined by crystal symmetry. An approximate (isotropic) treatment of cell e.s.d.'s is used for estimating e.s.d.'s involving l.s. planes.
Refinement. Refinement of *F*^2^ against ALL reflections. The weighted *R*-factor *wR* and goodness of fit *S* are based on *F*^2^, conventional *R*-factors *R* are based on *F*, with *F* set to zero for negative *F*^2^. The threshold expression of *F*^2^ > σ(*F*^2^) is used only for calculating *R*-factors(gt) *etc*. and is not relevant to the choice of reflections for refinement. *R*-factors based on *F*^2^ are statistically about twice as large as those based on *F*, and *R*- factors based on ALL data will be even larger.

### Fractional atomic coordinates and isotropic or equivalent isotropic displacement parameters (Å^2^)

**Table:**

	*x*	*y*	*z*	*U*_iso_*/*U*_eq_	
O1	0.57779 (16)	0.16291 (12)	0.51388 (7)	0.0404 (4)	
O19	0.65514 (15)	0.45154 (13)	0.40080 (8)	0.0407 (4)	
O3	0.73309 (14)	0.27588 (11)	0.43789 (8)	0.0390 (4)	
C8	0.2432 (2)	0.50423 (15)	0.40398 (10)	0.0252 (4)	
C10	−0.0015 (2)	0.63516 (16)	0.37433 (10)	0.0266 (4)	
H10	−0.1138	0.6545	0.3804	0.032*	
O20	0.33160 (19)	0.21986 (13)	0.54631 (9)	0.0584 (5)	
N14	0.02179 (17)	0.80169 (13)	0.29384 (9)	0.0307 (4)	
C12	0.2607 (2)	0.66959 (17)	0.32158 (11)	0.0317 (5)	
H12	0.3242	0.7124	0.2910	0.038*	
C9	0.0729 (2)	0.53919 (16)	0.40930 (10)	0.0259 (4)	
H9	0.0078	0.4944	0.4382	0.031*	
C11	0.0910 (2)	0.70529 (16)	0.32900 (10)	0.0266 (4)	
C4	0.6131 (2)	0.36263 (18)	0.42864 (11)	0.0310 (5)	
C13	0.3340 (2)	0.57427 (17)	0.35789 (11)	0.0309 (5)	
H13	0.4465	0.5550	0.3521	0.037*	
C7	0.3028 (2)	0.40397 (16)	0.44569 (10)	0.0275 (4)	
H7	0.2188	0.3745	0.4725	0.033*	
C15	0.1135 (2)	0.86883 (18)	0.24297 (11)	0.0386 (5)	
H15A	0.0311	0.9075	0.2070	0.046*	
H15B	0.1801	0.8159	0.2161	0.046*	
C5	0.4505 (2)	0.33863 (16)	0.45730 (10)	0.0292 (4)	
C6	0.4436 (3)	0.23885 (18)	0.50801 (11)	0.0377 (5)	
C2	0.6814 (2)	0.16029 (17)	0.45458 (12)	0.0363 (5)	
C18	−0.2946 (2)	0.7988 (2)	0.25955 (13)	0.0473 (6)	
H18A	−0.2752	0.8057	0.2085	0.071*	
H18B	−0.3967	0.8406	0.2673	0.071*	
H18C	−0.3077	0.7180	0.2716	0.071*	
C17	−0.1429 (2)	0.84969 (17)	0.30887 (12)	0.0349 (5)	
H17A	−0.1415	0.9336	0.3019	0.042*	
H17B	−0.1577	0.8348	0.3603	0.042*	
C21	0.8427 (3)	0.0962 (2)	0.48418 (14)	0.0551 (6)	
H21A	0.8967	0.1357	0.5274	0.083*	
H21B	0.9195	0.0943	0.4469	0.083*	
H21C	0.8146	0.0179	0.4971	0.083*	
C22	0.5872 (3)	0.1025 (2)	0.38720 (12)	0.0460 (6)	
H22A	0.5555	0.0247	0.3997	0.069*	
H22B	0.6600	0.0994	0.3484	0.069*	
H22C	0.4860	0.1465	0.3706	0.069*	
C16	0.2319 (3)	0.9596 (2)	0.28194 (14)	0.0533 (6)	
H16A	0.1656	1.0166	0.3047	0.080*	
H16B	0.2950	0.9971	0.2465	0.080*	
H16C	0.3102	0.9224	0.3194	0.080*	

### Atomic displacement parameters (Å^2^)

**Table:**

	*U*^11^	*U*^22^	*U*^33^	*U*^12^	*U*^13^	*U*^23^
O1	0.0464 (8)	0.0385 (9)	0.0367 (8)	0.0179 (6)	0.0058 (6)	0.0069 (7)
O19	0.0303 (7)	0.0382 (9)	0.0546 (10)	0.0026 (6)	0.0093 (6)	0.0078 (7)
O3	0.0274 (7)	0.0354 (8)	0.0538 (9)	0.0093 (6)	0.0033 (6)	0.0008 (7)
C8	0.0267 (9)	0.0252 (10)	0.0236 (10)	0.0011 (7)	0.0025 (7)	−0.0030 (8)
C10	0.0229 (9)	0.0296 (11)	0.0276 (10)	0.0014 (7)	0.0039 (7)	−0.0005 (9)
O20	0.0643 (10)	0.0510 (11)	0.0666 (11)	0.0218 (8)	0.0352 (9)	0.0286 (9)
N14	0.0289 (8)	0.0294 (9)	0.0341 (10)	0.0030 (7)	0.0042 (7)	0.0077 (7)
C12	0.0287 (9)	0.0320 (12)	0.0357 (12)	−0.0004 (8)	0.0096 (8)	0.0068 (9)
C9	0.0263 (9)	0.0292 (11)	0.0229 (10)	−0.0026 (7)	0.0058 (7)	−0.0004 (8)
C11	0.0291 (9)	0.0257 (10)	0.0241 (10)	0.0014 (8)	−0.0003 (7)	−0.0027 (8)
C4	0.0285 (10)	0.0321 (12)	0.0315 (11)	0.0051 (8)	0.0000 (8)	−0.0043 (10)
C13	0.0243 (9)	0.0335 (11)	0.0359 (12)	0.0028 (8)	0.0077 (8)	0.0029 (9)
C7	0.0292 (9)	0.0266 (11)	0.0279 (11)	0.0007 (8)	0.0082 (7)	−0.0034 (9)
C15	0.0417 (11)	0.0358 (12)	0.0389 (12)	0.0026 (9)	0.0070 (9)	0.0156 (10)
C5	0.0303 (9)	0.0279 (11)	0.0293 (11)	0.0026 (8)	0.0033 (7)	−0.0022 (9)
C6	0.0430 (11)	0.0346 (12)	0.0364 (12)	0.0103 (9)	0.0086 (9)	0.0034 (10)
C2	0.0360 (11)	0.0324 (12)	0.0403 (13)	0.0108 (9)	0.0033 (9)	0.0005 (10)
C18	0.0347 (11)	0.0472 (14)	0.0580 (15)	0.0033 (9)	−0.0029 (10)	0.0115 (12)
C17	0.0342 (10)	0.0286 (11)	0.0421 (12)	0.0069 (8)	0.0057 (8)	0.0037 (10)
C21	0.0446 (12)	0.0548 (16)	0.0634 (17)	0.0220 (11)	−0.0038 (11)	0.0016 (13)
C22	0.0522 (12)	0.0397 (14)	0.0441 (14)	0.0123 (10)	−0.0023 (10)	−0.0057 (11)
C16	0.0518 (13)	0.0419 (14)	0.0670 (17)	−0.0090 (10)	0.0103 (11)	0.0091 (12)

### Geometric parameters (Å, º)

**Table:**

O1—C6	1.363 (2)	C7—H7	0.9300
O1—C2	1.426 (2)	C15—C16	1.514 (3)
O19—C4	1.201 (2)	C15—H15A	0.9700
O3—C4	1.368 (2)	C15—H15B	0.9700
O3—C2	1.429 (2)	C5—C6	1.473 (3)
C8—C9	1.413 (2)	C2—C22	1.506 (3)
C8—C13	1.413 (2)	C2—C21	1.509 (3)
C8—C7	1.424 (2)	C18—C17	1.519 (3)
C10—C9	1.366 (2)	C18—H18A	0.9600
C10—C11	1.413 (3)	C18—H18B	0.9600
C10—H10	0.9300	C18—H18C	0.9600
O20—C6	1.205 (2)	C17—H17A	0.9700
N14—C11	1.357 (2)	C17—H17B	0.9700
N14—C15	1.457 (2)	C21—H21A	0.9600
N14—C17	1.463 (2)	C21—H21B	0.9600
C12—C13	1.367 (3)	C21—H21C	0.9600
C12—C11	1.418 (2)	C22—H22A	0.9600
C12—H12	0.9300	C22—H22B	0.9600
C9—H9	0.9300	C22—H22C	0.9600
C4—C5	1.463 (2)	C16—H16A	0.9600
C13—H13	0.9300	C16—H16B	0.9600
C7—C5	1.377 (2)	C16—H16C	0.9600
			
C6—O1—C2	117.50 (15)	O20—C6—C5	125.78 (18)
C4—O3—C2	119.35 (14)	O1—C6—C5	117.29 (17)
C9—C8—C13	115.46 (16)	O1—C2—O3	110.09 (15)
C9—C8—C7	116.56 (16)	O1—C2—C22	110.63 (16)
C13—C8—C7	127.97 (16)	O3—C2—C22	111.13 (17)
C9—C10—C11	120.46 (16)	O1—C2—C21	105.88 (17)
C9—C10—H10	119.8	O3—C2—C21	106.15 (16)
C11—C10—H10	119.8	C22—C2—C21	112.74 (18)
C11—N14—C15	121.78 (15)	C17—C18—H18A	109.5
C11—N14—C17	122.20 (15)	C17—C18—H18B	109.5
C15—N14—C17	115.86 (15)	H18A—C18—H18B	109.5
C13—C12—C11	122.18 (17)	C17—C18—H18C	109.5
C13—C12—H12	118.9	H18A—C18—H18C	109.5
C11—C12—H12	118.9	H18B—C18—H18C	109.5
C10—C9—C8	123.61 (16)	N14—C17—C18	113.34 (17)
C10—C9—H9	118.2	N14—C17—H17A	108.9
C8—C9—H9	118.2	C18—C17—H17A	108.9
N14—C11—C10	122.12 (16)	N14—C17—H17B	108.9
N14—C11—C12	121.31 (16)	C18—C17—H17B	108.9
C10—C11—C12	116.57 (16)	H17A—C17—H17B	107.7
O19—C4—O3	116.57 (16)	C2—C21—H21A	109.5
O19—C4—C5	127.27 (17)	C2—C21—H21B	109.5
O3—C4—C5	116.09 (17)	H21A—C21—H21B	109.5
C12—C13—C8	121.68 (16)	C2—C21—H21C	109.5
C12—C13—H13	119.2	H21A—C21—H21C	109.5
C8—C13—H13	119.2	H21B—C21—H21C	109.5
C5—C7—C8	137.58 (17)	C2—C22—H22A	109.5
C5—C7—H7	111.2	C2—C22—H22B	109.5
C8—C7—H7	111.2	H22A—C22—H22B	109.5
N14—C15—C16	112.95 (18)	C2—C22—H22C	109.5
N14—C15—H15A	109.0	H22A—C22—H22C	109.5
C16—C15—H15A	109.0	H22B—C22—H22C	109.5
N14—C15—H15B	109.0	C15—C16—H16A	109.5
C16—C15—H15B	109.0	C15—C16—H16B	109.5
H15A—C15—H15B	107.8	H16A—C16—H16B	109.5
C7—C5—C4	126.92 (18)	C15—C16—H16C	109.5
C7—C5—C6	115.10 (16)	H16A—C16—H16C	109.5
C4—C5—C6	117.86 (16)	H16B—C16—H16C	109.5
O20—C6—O1	116.90 (18)		
			
C11—C10—C9—C8	0.8 (3)	C8—C7—C5—C4	4.4 (4)
C13—C8—C9—C10	−1.4 (3)	C8—C7—C5—C6	−179.8 (2)
C7—C8—C9—C10	178.19 (17)	O19—C4—C5—C7	13.2 (3)
C15—N14—C11—C10	−175.52 (17)	O3—C4—C5—C7	−170.14 (18)
C17—N14—C11—C10	9.3 (3)	O19—C4—C5—C6	−162.49 (19)
C15—N14—C11—C12	4.1 (3)	O3—C4—C5—C6	14.2 (2)
C17—N14—C11—C12	−171.03 (17)	C2—O1—C6—O20	160.50 (19)
C9—C10—C11—N14	−179.51 (17)	C2—O1—C6—C5	−21.3 (2)
C9—C10—C11—C12	0.8 (3)	C7—C5—C6—O20	−10.3 (3)
C13—C12—C11—N14	178.48 (18)	C4—C5—C6—O20	166.0 (2)
C13—C12—C11—C10	−1.8 (3)	C7—C5—C6—O1	171.75 (17)
C2—O3—C4—O19	−165.89 (17)	C4—C5—C6—O1	−12.0 (3)
C2—O3—C4—C5	17.1 (2)	C6—O1—C2—O3	50.5 (2)
C11—C12—C13—C8	1.3 (3)	C6—O1—C2—C22	−72.7 (2)
C9—C8—C13—C12	0.4 (3)	C6—O1—C2—C21	164.84 (18)
C7—C8—C13—C12	−179.18 (18)	C4—O3—C2—O1	−48.8 (2)
C9—C8—C7—C5	179.1 (2)	C4—O3—C2—C22	74.2 (2)
C13—C8—C7—C5	−1.4 (4)	C4—O3—C2—C21	−162.94 (17)
C11—N14—C15—C16	−85.9 (2)	C11—N14—C17—C18	−89.7 (2)
C17—N14—C15—C16	89.6 (2)	C15—N14—C17—C18	94.8 (2)

### Hydrogen-bond geometry (Å, º)

**Table:**

*D*—H···*A*	*D*—H	H···*A*	*D*···*A*	*D*—H···*A*
C13—H13···O19	0.93	2.13	2.915 (2)	141
C17—H17*B*···O20^i^	0.97	2.39	3.268 (3)	151

Symmetry code: (i) −*x*, −*y*+1, −*z*+1.
